# Healthcare resources attributable to child tobacco smoke exposure

**DOI:** 10.1371/journal.pone.0247179

**Published:** 2021-02-23

**Authors:** Ashley L. Merianos, Roman A. Jandarov, Judith S. Gordon, Michael S. Lyons, E. Melinda Mahabee-Gittens

**Affiliations:** 1 School of Human Services, University of Cincinnati, Cincinnati, Ohio, United States of America; 2 Center for Addiction Research, College of Medicine, University of Cincinnati, Cincinnati, Ohio, United States of America; 3 Department of Environmental and Public Health Sciences, Division of Biostatistics and Bioinformatics, College of Medicine, University of Cincinnati, Cincinnati, Ohio, United States of America; 4 College of Nursing, The University of Arizona, Tucson, Arizona, United States of America; 5 Department of Emergency Medicine, College of Medicine, University of Cincinnati, Cincinnati, Ohio, United States of America; 6 Division of Emergency Medicine, Cincinnati Children’s Hospital Medical Center, College of Medicine, University of Cincinnati, Cincinnati, Ohio, United States of America; University of Calfornia San Francisco, UNITED STATES

## Abstract

**Background:**

Tobacco smoke exposure (TSE) places an economic toll on the U.S. healthcare system. There is a gap in the literature on pediatric emergency department (ED) and urgent care related healthcare costs and utilization specific to tobacco smoke-exposed patients. The objectives were to assess pediatric ED visits, urgent care visits and hospital admissions longitudinally, and baseline visit costs among tobacco smoke-exposed children (TSE group) relative to unexposed children (non-TSE group).

**Methods and findings:**

We conducted a retrospective study using electronic medical records of 380 children ages 0–17 years in the TSE group compared to 1,140 in the non-TSE group propensity score matched via nearest neighbor search by child age, sex, race, and ethnicity. Linear and Poisson regression models were used. Overall, children had a mean of 0.19 (SE = 0.01) repeat visits within 30-days, and 0.69 (SE = 0.04) pediatric ED visits and 0.87 (SE = 0.03) urgent care visits over 12-months following their baseline visit. The percent of children with ≥ 1 urgent care visit was higher among the TSE group (52.4%) than the non-TSE group (45.1%, *p* = 0.01). Children in the TSE group (*M* = $1,136.97, SE = 76.44) had higher baseline pediatric ED visit costs than the non-TSE group (*M* = $1,018.96, SE = 125.51, *p* = 0.01). Overall, children had 0.08 (SE = 0.01) hospital admissions over 12-months, and the TSE group (*M* = 0.12, SE = 0.02) had higher mean admissions than the non-TSE group (*M* = 0.06, SE = 0.01, *p* = 0.02). The child TSE group was at 1.85 times increased risk of having hospital admissions (95% CI = 1.23, 2.79, *p* = 0.003) than the non-TSE group.

**Conclusions:**

Tobacco smoke-exposed children had higher urgent care utilization and hospital admissions over 12-months, and higher pediatric ED costs at baseline. Pediatric ED visits, urgent care visits, and hospitalizations may be opportune times for initiating tobacco control interventions, which may result in reductions of preventable acute care visits.

## Introduction

Tobacco smoke exposure (TSE) substantially contributes to increased U.S. healthcare expenditures and loss of productivity [1]. Children are typically subjected to higher exposure to tobacco smoke than adults due to developmental physiology and age-related patterns [2], and approximately 40% of children remain exposed nationwide [3, 4]. Child TSE includes exposure to secondhand smoke where the child inhales freshly emitted smoke from a lit tobacco product, and exposure to thirdhand smoke pollution where the child inhales, ingests, and/or dermally absorbs residue that remains in environments after tobacco use has ceased [5]. Children have physiological characteristics that result in higher exposure to tobacco smoke toxicants, which can result in greater TSE inhalation among children compared to adults in the same environment [2]. Child TSE-related morbidity includes but is not limited to: respiratory-related problems and infections, asthma and related exacerbations, ear infections, and sudden infant death syndrome [1, 6]. Additionally, young children spend over 90% of their time indoors, which increases their exposure to tobacco smoke [2]. Children who live with smokers have lower prevalence of smoke-free homes than children who live with nonsmokers [7].

Child TSE places an economic toll on the U.S. healthcare system. While cigarette smoking recently reached a national record low [8], smoking-attributable healthcare costs still account for approximately 5–14% of overall healthcare expenditures nationwide [1]. Loss of productivity attributed specifically to TSE-related premature deaths is estimated to be around $6.6 billion [9], but this amount is largely underestimated since it does not consider the productivity loss value of TSE-related morbidity. Direct medical and nonmedical costs and productivity loss for biochemically validated TSE among nonsmoking children ages 3–19 years residing in public housing are estimated at about $77 million and $19 million, respectively, amounting to a total societal cost of $96 million [10]. Smoking in children’s homes has been associated with an additional $117 in healthcare costs for respiratory-related conditions among children ages 0–4 years, and an increased likelihood of having an emergency department (ED) visit and hospital stay [11]. The available literature has not specifically examined TSE and healthcare costs related to pediatric ED and urgent care visits [10–13]. Prior research specific to the healthcare costs of ED visits only assessed patients with asthma and did not take into consideration other diagnoses [14].

Our objective was to assess baseline pediatric ED visit costs, urgent care visit costs, and hospital admissions costs attributable to TSE among tobacco smoke-exposed children (TSE group) relative to unexposed children (non-TSE group). We hypothesized that tobacco smoke-exposed children would have higher healthcare expenditures during the baseline pediatric ED or urgent care visits compared to unexposed children. Longitudinally, we examined the associations between child TSE and pediatric ED visits, urgent care visits, and hospital admissions 12-months following the baseline pediatric ED and urgent care visits. We posited the TSE group would be more likely to have pediatric ED visits, urgent care visits, and hospitalizations over 12-months than the non-TSE group.

## Materials and methods

### Study design

We conducted an Institutional Review Board (IRB)-approved retrospective study using electronic medical records (EMRs) from a cohort of 1,520 children previously identified by two IRB-studies conducted at a U.S. children’s hospital. The hospital’s pediatric ED and urgent care are among the busiest across the nation with over 150,000 annual patient encounters. We obtained cross-sectional cost data and longitudinal healthcare visit data from children’s EMRs. This EMR review including identifiable data was reviewed and approved using expedited review procedures under federal regulation 45 CFR 46.110 by the University of Cincinnati IRB (#2017–2081). The IRB waived the requirement to obtain informed consent in accordance with 45 CFR 46.116 and the requirement to obtain an authorization for the use of protected health information in accordance with 45 CFR 164.512.

#### Child TSE group

Our TSE group was comprised of 380 children ages 0–17 years who were exposed to tobacco smoke and presented to the pediatric ED or urgent care. This group was originally enrolled in the Healthy Families smoking cessation randomized controlled trial (RCT) registered as NCT-01193374 on www.clinicaltrials.gov [15]. This RCT was reviewed and approved by the Cincinnati Children’s Hospital Medical Center IRB (#2015–1914). To be enrolled into Healthy Families, children had to meet the following eligibility criteria: presented with a chief complaint that was potentially TSE-related as outlined by the U.S. Surgeon General [6, 16], were triaged in the non-urgent or urgent category, were deemed clinically stable as confirmed by pediatric ED and urgent care practitioners prior to approaching the families about participation; had a parent present at the visit who self-reported smoking combustible tobacco products; and the child self-reported not using any combustible or non-combustible tobacco products. Parents who exclusively used electronic cigarettes (e-cigarettes), smokeless tobacco, or smoking cessation medications at baseline were ineligible for the study. We excluded parents who exclusively used e-cigarettes due to the low prevalence of exclusive e-cigarette users in our population, but included those who reported concurrent use with combustible tobacco products. Five percent (*n* = 20) of parents of children in the TSE group concurrently used e-cigarettes with combustible tobacco products. Potential TSE-related chief complaints included cough, congestion, difficulty breathing, wheezing, ear pain, and sore throat.

#### Non-TSE group

Our non-TSE group was comprised of 1,140 children ages 0–17 years who were unexposed to tobacco smoke and presented to the pediatric ED or urgent care. This group was originally enrolled in a study that tested a clinical decision support system that facilitated tobacco screening and counseling among adult tobacco users in the pediatric ED and urgent care setting registered as NCT-02489708 on www.clinicaltrials.gov [17, 18]. This study was reviewed and approved by the Cincinnati Children’s Hospital Medical Center IRB (#2014–5592). Children had to meet the following eligibility criteria to be considered unexposed to tobacco smoke: parents reported they did not use tobacco, and parents reported the child did not live with anyone who used tobacco.

### Study setting and participants

This study matched the TSE and non-TSE groups using propensity scores via nearest neighbor search while matching on the child’s age, sex, race, and ethnicity. The ratio of one child in the TSE group to three children in the non-TSE group was preferred to detect between-group differences. We set parameters to ensure the groups presented at the same pediatric ED and urgent care locations and that visit dates were within a 12-month period.

### Measures

#### Longitudinal healthcare visits

We extracted longitudinal EMR data on healthcare visits following the baseline visit. EMR data are optimal for healthcare visit and cost data in order to reduce biases related to self-report limitations such as recall and reporting issues [19]. Measured prospectively following the baseline pediatric ED and urgent care visits, healthcare visit data assessed were: 1) repeat visits to the pediatric ED or urgent care within 30 days; and 2) pediatric ED visits, 3) urgent care visits, and 4) hospital admissions over 12-months.

#### Baseline healthcare costs

We obtained cross-sectional healthcare costs for the baseline pediatric ED and urgent care visits only, and included the following cost data as outcome variables: pediatric ED costs and urgent care costs. For children who were admitted to the hospital during their baseline pediatric ED or urgent care visits, we also included baseline admissions costs. Baseline costs were defined as pediatric ED, urgent care, and hospital admissions billed charges. Data were not available on out-of-pocket costs for the patients or healthcare reimbursement costs the hospital received for providing the medical services.

#### Child characteristics

We extracted sociodemographic data from EMRs and matched groups based on child age, sex, race (white, black, and other), and ethnicity (non-Hispanic, Hispanic, and unknown). Other race included Asian, other race not specified, and multiple races. We abstracted information on insurance type (private or Medicaid/self-pay) and baseline visit disposition (discharge to home or admit to hospital). Of those in the Medicaid/self-pay group, most had Medicaid (*n* = 1,111) and only 49 were self-pay. We extracted data on past medical history (PMH) of respiratory illnesses (asthma, bronchiolitis, and pneumonia), PMH of prematurity, and the baseline visit primary discharge diagnosis.

### Data analysis

First, we matched the child TSE and non-TSE groups using propensity score matching via nearest neighbor search to establish groups that shared similar characteristics of child age, sex, race, and ethnicity. We calculated descriptive statistics including frequencies to describe the characteristics overall, and by TSE group.

For each healthcare visit outcome variable (e.g., repeat visits), we calculated three separate measures: overall mean visits/admissions per child irrespective of having positive utilization; mean visits/admissions per child who had positive utilization (i.e., ≥ 1 visit/admission of the outcome of interest); and the percent of children who had positive utilization (i.e., ≥ 1 visit/admission). We conducted linear regression analyses to assess the differences between the matched groups and mean number of visits/admissions overall, and among those who had positive utilization. We conducted chi-square tests to assess the differences between the matched groups and the percent of children with positive utilization.

To further examine patterns, we conducted separate adjusted Poisson regression models to assess the associations between TSE groups and healthcare visit outcome variables. We included child age, sex, race, ethnicity, and insurance type in the models, and present adjusted relative risk (aRR) ratios and corresponding 95% confidence intervals (95% CIs).

To assess TSE group differences based on baseline visit costs, we conducted four separate multiple regression models. We also included the demographic variables and insurance type in the models, and present betas (β) and 95% CIs. While assessing the data for normality prior to analyses, we identified two outliers for hospital admissions that were ≥ $30,000, which were excluded from cost analyses. Statistical significance was set at *p* < 0.05 and all analyses were two-sided and performed using R statistical software (version 3.5.1) [20].

## Results

Mean child age (SD) was 4.9 (0.1) years, about half (50.5%) were female, and 55.6% were black followed by 35.9% white and 8.5% other race. Table 1. The majority of children were non-Hispanic (98.4%) and had Medicaid insurance/self-pay (73.2%). There were no differences based on age, sex, race, and ethnicity since the child TSE and non-TSE groups were matched on these demographics prior to analyses.

**Table 1:** Child characteristics overall and by tobacco smoke exposure group status among nonsmoking children ages 0–17 years.

	Overall	Non-TSE Group	TSE Group
Characteristic	(N = 1,520)	(*n* = 1,140)	(*n* = 380)
**Child Sociodemographics**			
	***n* (%)^a^**	***n* (%)^a^**	***n* (%)^a^**
**Age**			
0 years	234 (15.4)	138 (12.1)	96 (25.3)
1 year	313 (20.6)	260 (22.8)	53 (13.5)
2–4 years	345 (22.7)	258 (22.6)	87 (22.9)
5–9 years	346 (22.8)	268 (23.5)	78 (20.5)
10–17 years	282 (18.5)	216 (19.0)	66 (17.4)
**Sex**			
Male	753 (49.5)	564 (49.5)	189 (49.7)
Female	767 (50.5)	576 (50.5)	191 (50.3)
**Race**			
White	537 (35.9)	405 (35.9)	132 (35.6)
Black	832 (55.6)	620 (55.0)	212 (57.1)
Other	129 (8.5)	102 (9.1)	27 (7.3)
**Ethnicity**			
Non-Hispanic	1496 (98.4)	1126 (98.8)	370 (97.4)
Hispanic	19 (1.3)	14 (1.2)	5 (1.3)
Unknown	5 (0.3)	0 (0.0)	5 (1.3)
**Insurance Type**			
Commercial	407 (26.8)	379 (33.3)	28 (7.4)
Medicaid/Self-pay	1111 (73.2)	759 (66.7)	352 (92.6)
**Child Characteristics at Baseline Visit**			
**PMH of Respiratory Illness**^**c**^			
No	1,277 (84.0)	970 (85.1)	307 (80.8)
Yes	243 (16.0)	170 (14.9)	73 (19.2)
**PMH of Prematurity**			
No	1,433 (94.3)	1,081 (94.8)	352 (92.6)
Yes	87 (5.7)	59 (5.2)	28 (7.4)
**Disposition at Baseline Visit**			
Discharge to Home	1,485 (97.7)	1,131 (99.2)	354 (93.2)
Admit to Hospital	35 (2.3)	9 (0.8)	26 (6.8)
**Primary Discharge Diagnosis at Baseline Visit**			
Non-TSE Related	688 (45.3)	614 (53.9)	74 (19.5)
Asthma	47 (3.1)	18 (1.6)	29 (7.6)
Bronchiolitis	44 (2.9)	16 (1.4)	28 (7.4)
Pneumonia	12 (0.8)	8 (0.7)	4 (1.0)
Upper Respiratory Infection/Croup	431 (28.3)	261 (22.9)	170 (44.7)
Otitis Media	240 (15.8)	177 (15.5)	63 (16.6)
Conjunctivitis	58 (3.8)	46 (4.0)	12 (3.2)

Abbreviation: TSE, tobacco smoke exposure; PMH, past medical history.

^a^Percent refers to column percent. Missing values excluded.

^b^Respiratory illness includes asthma, bronchiolitis, and pneumonia.

Sixteen percent of children had history of respiratory illness and 5.7% had history of prematurity. Most children were discharged to home (97.7%) from the baseline visit, and the top three discharge diagnoses were non-TSE related (45.3%), upper respiratory infection or croup (28.3%), and otitis media (15.8%). See Table 1.

### Healthcare visit patterns over 12-months

Overall, children had a mean of 0.19 (SE = 0.01) repeat visits within 30 days, and 0.69 (SE = 0.04) pediatric ED visits and 0.87 (SE = 0.03) urgent care visits over 12-months following the baseline pediatric ED and urgent care visits. Table 2. Of children with positive utilization, they had a mean of 1.12 (SE = 0.02) repeat visits, 1.78 (SE = 0.07) pediatric ED visits, and 1.86 (SE = 0.05) urgent care visits. No between-group differences were found between mean visits overall, and by positive utilization. The percent of children having positive utilization of at least one repeat visit, pediatric ED visit, or urgent care visit was 16.8%, 38.5%, and 46.9%, respectively. Chi-square results indicated the percent of children with ≥ 1 urgent care visit was higher among the TSE group (52.4%) than the non-TSE group (45.1%, *p* = 0.01). See Table 2.

**Table 2:** Healthcare visit and hospitalization patterns over 12-months by tobacco smoke exposure group status among nonsmoking children ages 0–17 years.

Healthcare Visit Variable	Overall^a^	Non-TSE Group^b^	TSE Group^b^	*P*-value
**Repeat Visits within 30-Days from Baseline Visit**				
Mean among all patients, *M* (SE)	0.19 (0.01)	0.19 (0.01)	0.19 (0.02)	0.99
Mean among patients with ≥1 repeat visit, *M* (SE)	1.12 (0.02)	1.11 (0.03)	1.16 (0.05)	0.37
Percent of patients with ≥1 repeat visit, *n* (%)	256 (16.8)	194 (17.0)	62 (16.3)	0.75
**Pediatric ED Visits over 12-Months following Baseline Visit**				
Mean among all patients, *M* (SE)	0.69 (0.04)	0.66 (0.04)	0.77 (0.06)	0.20
Mean among patients with ≥1 pediatric ED visit, *M* (SE)	1.78 (0.07)	1.77 (0.09)	1.82 (0.10)	0.76
Percent of patients with ≥1 pediatric ED visit, *n* (%)	585 (38.5)	425 (37.3)	160 (42.1)	0.09
**Urgent Care Visits over 12-Months following Baseline Visit**				
Mean among all patients, *M* (SE)	0.87 (0.03)	0.84 (0.04)	0.98 (0.07)	0.08
Mean among patients with ≥1 urgent care visit, *M* (SE)	1.86 (0.05)	1.86 (0.06)	1.86 (0.08)	0.99
Percent of patients with ≥1 urgent care visit, *n* (%)	713 (46.9)	514 (45.1)	199 (52.4)	0.01
**Hospital Admissions over 12-Months following Baseline Visit**				
Mean among all patients, *M* (SE)	0.08 (0.01)	0.06 (0.01)	0.12 (0.02)	0.02
Mean among patients with ≥1 admission, *M* (SE)	1.27 (0.08)	1.18 (0.08)	1.47 (0.18)	0.10
Percent of patients with ≥1 admission, *n* (%)	91 (6.0)	61 (5.4)	30 (7.9)	0.07

Abbreviation: TSE, tobacco smoke exposure; *M*, mean; SE, standard error; ED, emergency department.

N = 1,520.

^a^Percent of patients percent refers to row percent

^b^Percent of patients percent refers to column percent.

Children had 0.08 (SE = 0.01) hospital admissions over 12-months, and linear regression results indicated the TSE group (*M* = 0.12, SE = 0.02) had higher mean admissions than the non-TSE group (*M* = 0.06, SE = 0.01, *p* = 0.02). The percent of children with ≥ 1 hospital admission was 6.0% with a mean of 1.27 (SE = 0.08) admissions. See Table 2.

Poisson regression results indicated that after adjustment for child age, sex, race, ethnicity, and insurance type, there were no differences between TSE groups based on repeat visits, pediatric ED visits, or urgent care visits. Table 3. The child TSE group was at 1.85 times increased risk of having hospital admissions (95% CI = 1.23,2.79, *p* = 0.003) than the non-TSE group.

**Table 3:** Tobacco smoke exposure group status and healthcare utilization patterns over 12-months among nonsmoking children ages 0–17 years.

	Repeat Visits within 30-Days	Pediatric ED Visits over 12-Months	Urgent Care Visits over 12-Months	Hospital Admissions over 12-Months
	aRR (95% CI)^a^	aRR (95% CI)^a^	aRR (95% CI)^a^	aRR (95% CI)^a^
**TSE Group**				
Non-TSE	Ref	Ref	Ref	Ref
TSE	0.88 (0.66, 1.17)	0.97 (0.84, 1.12)	1.01 (0.89, 1.15)	1.85 (1.23, 2.79)**
**Age**				
0 years	Ref	Ref	Ref	Ref
1 year	1.13 (0.79, 1.61)	0.84 (0.70, 1.02)	0.87 (0.74, 1.02)	1.35 (0.80, 2.28)
2–4 years	0.65 (0.44, 0.96)*	0.63 (0.52, 0.77)***	0.64 (0.54, 0.75)***	0.55 (0.29, 1.04)
5–9 years	0.62 (0.41, 0.93)*	0.51 (0.42, 0.64)***	0.53 (0.44, 0.63)***	0.36 (0.17, 0.78)**
10–17 years	0.86 (0.58, 1.27)	0.83 (0.68, 1.01)	0.45 (0.37, 0.55)***	1.11 (0.62, 1.99)
**Sex**				
Male	Ref	Ref	Ref	Ref
Female	1.09 (0.86, 1.38)	1.07 (0.94,1.21)	1.07 (0.95, 1.19)	0.57 (0.38, 0.84)**
**Race**				
White	Ref	Ref	Ref	Ref
Black	0.97 (0.73, 1.29)	0.74 (0.64, 0.85)***	1.26 (1.10, 1.44)***	0.48 (0.31, 0.75)**
Other	1.43 (0.96, 2.12)	1.2 (0.98, 1.47)	1.22 (0.99, 1.51)	1.49 (0.87, 2.54)
**Ethnicity**				
Non-Hispanic	Ref	Ref	Ref	Ref
Hispanic	1.34 (0.43, 4.23)	0.83 (0.39, 1.75)	0.78 (0.39, 1.56)	-
Unknown	1.25 (0.17, 9.08)	-	1.73 (0.77, 3.90)	-
**Insurance Type**				
Commercial	Ref	Ref	Ref	Ref
Medicaid/Self-pay	1.59 (1.15, 2.20)**	2.15 (1.79, 2.57)***	1.48 (1.27, 1.74)***	1.67 (0.99, 2.80)

Abbreviations: TSE, tobacco smoke exposure; ED, emergency department; aRR, adjusted relative risk; CI, confidence interval; Ref, reference group.

N = 1,520.

*** *p* < 0.001

** *p* < 0.01

* *p* < 0.05.

^a^Poisson regression analysis including TSE group, age, sex, race, ethnicity, and insurance type.

### Baseline healthcare costs

Multiple regression results indicated children in the TSE group (*M* = $1,136.97, SE = 76.44) had higher baseline pediatric ED costs than the non-TSE group (*M* = $1,018.96, SE = 125.51, *p* = 0.01) while controlling for child age, sex, race, ethnicity, and insurance type. Table 4. No between-group differences were found based on urgent care or hospital admissions costs.

**Table 4:** Tobacco smoke exposure group status and pediatric ED visit, urgent care visit, and hospitalization costs from baseline among nonsmoking children ages 0–17 years.

	Pediatric ED Costs from Baseline Visit (*n* = 140)		Urgent Care Costs from Baseline Visit (*n* = 1,408)		Hospital Admissions Costs from Baseline Visit (*n* = 33)	
	*M* (SE)	β (95% CI)^a^	*M* (SE)	β (95% CI)^a^	*M* (SE)	β (95% CI)^a^
**TSE Group**						
Non-TSE	1,018.96 (125.51)	Ref	308.53 (8.31)	Ref	7,848.43 (2,432.94)	Ref
TSE	1,136.97 (76.44)	386.89 (81.49, 692.29)*	220.55 (12.55)	1.44 (-31.37, 34.25)	9,547.36 (1,211.24)	2,265.20 (-7,342.24, 11,872.63)
**Age**						
0 years	834.92 (111.18)	Ref	275.58 (16.77)	Ref	8,641.17 (2,578.35)	Ref
1 year	1,155.52 (142.89)	315.48 (-71.28, 702.23)	271.33 (16.75)	7.37 (-35.25, 49.99)	8,775.38 (1,599.04)	-421.91 (-6,851.02, 6,007.19)
2–4 years	1,103.18 (138.08)	341.79 (-51.42, 735)	293.36 (15.47)	18.46 (-22.86, 59.79)	8,502.2 (2,736.72)	-1,222.39 (-11,328.37, 8,883.58)
5–9 years	1,375.32 (185.75)	503.07 (125.59, 880.56)*	291.97 (14.48)	25.7 (-16.41, 67.80)	11,719.12 (2,893.81)	308.18 (-7,750.81, 8,367.18)
10–17 years	1,155.45 (138.95)	142.62 (-210.75, 495.99)	325.52 (16.69)	36.71 (-7.48, 80.89)	9,117.59 (2,873.99)	152.68 (-9,057.06, 9,362.42)
**Sex**						
Male	1,111.96 (86.86)	Ref	290.4 (10.55)	Ref	6,969.81 (1,178.66)	Ref
Female	1,113.04 (98.6)	-48.18 (-287.26, 190.9)	292.91 (9.79)	-2.26 (-27.01, 22.49)	11,034.63 (1,608.00)	4,336.44 (-327.26, 9,000.13)
**Race**						
White	1,162.15 (112.43)	Ref	378.21 (14.37)	Ref	7,532.69 (1309.86)	Ref
Black	1,058.2 (88.38)	1.87 (-262.72, 266.45)	237.83 (7.9)	-13.47 (-43.44, 16.49)	10,600.37 (1,504.12)	4,581.10 (-1,444.99, 10,607.18)
Other	1,127.79 (178.37)	-49.65 (-465.45, 366.14)	296.50 (24.95)	-23.36 (-70.02, 23.30)	10,443.71 (5,558.39)	1,608.62 (-6,603.54, 9,820.78)
**Ethnicity**						
Non-Hispanic	1,103.87 (66.38)	Ref	291.80 (7.28)	Ref	9,309.76 (1,100.87)	Ref
Hispanic	1,244.34 (0.00)	1,276.94 (1,273.42, 1,280.47)	313.18 (38.69)	41.16 (-86.12, 168.45)	0 (0.00)	-8,956.45 (-8,954.2, -8,958.71)
Unknown	2,175.31 (0.00)	709.62 (709.47, 709.76)	145.39 (29.02)	-73.69 (-302.78, 155.41)	5,257.96 (0.00)	7,780.88 (7,780.36, 7,781.39)
**Insurance Type**						
Commercial	1,788.13 (185.71)	Ref	517.34 (15.45)	Ref	9,910.4 (3,196.37)	Ref
Medicaid/Self-pay	958.44 (60.70)	-939.09 (-1,269.40, -608.77)***	204.32 (6.09)	-308.78 (-340.21, -277.34)***	9,057.8 (1,156.25)	-3,600.84 (-12,640.05, 5,438.38)

Abbreviations: TSE, tobacco smoke exposure; ED, emergency department; *M*, mean; SE, standard error; CI, confidence interval; Ref, reference group.

*** *p* < 0.001

* *p* < 0.05.

*n* = 1,518 after removing two outliers ≥ $30,000.

^a^Multiple regression analysis including TSE group, age, sex, race, ethnicity, and insurance type.

## Discussion

This study indicates that children exposed to tobacco smoke had higher urgent care utilization and hospital admissions than unexposed children matched by age, sex, race and ethnicity. Specifically, over half of the TSE group had positive urgent care utilization over 12-months following the baseline pediatric ED and urgent care visits, which was significantly higher than the non-TSE group. While not statistically significant, the TSE group had a higher percent who had positive pediatric ED utilization (42.1%) than the non-TSE group (37.3%). Our findings expand upon national research that indicated tobacco smoke-exposed children had higher ED visits (24%) compared to unexposed children (18%) [13].

Prior research indicates that child and adolescent TSE is associated with higher ED and urgent care visits, but did not assess these as distinct settings [21, 22]. Considering these settings individually is important since urgent cares can be utilized to lighten the non-emergent care burden placed on increasingly overcrowded EDs, which in turn, may lead to healthcare cost savings [23, 24]. Urgent cares may be an important setting in which TSE interventions can be conducted using the EMR to briefly screen and counsel parental smokers without disrupting clinical flow [25, 26]. A meta-analysis of ED-initiated tobacco control RCTs concluded that these efforts are effective in promoting repeated tobacco abstinence [27]. Given the recent rise in the number of urgent cares nationwide, this will be an important new locus of intervention [28].

We found that the child TSE group had significantly higher pediatric ED costs than the non-TSE group after adjustment for sociodemographics. The average pediatric ED costs were $1,137 for the TSE group, which was $118 higher than the average cost for the non-TSE group. An older study found low-income, asthmatic children who lived with ≥ 1 smoker had higher mean TSE-attributable ED visits with an estimated annual cost of $92 per child than children who did not live with smokers, but no differences were found based on hospitalizations [14]. Other work using 2007 Medicaid expenditure data found that children living with a smoker did not have higher overall costs, but did have significantly higher costs of about $10–13 more per child specific to ED annual expenditures compared to children living with nonsmokers [12]. An analysis of National Health Interview Survey 2010 data revealed that TSE was associated with higher ED visits among children ages 3–14 years, with an excess of 101,570 ED visits and $62.9 million in annual healthcare expenditures [13]. Our results show that mean baseline pediatric ED costs for the child TSE group are disproportionately higher compared to the other studies [23, 29, 30]. However, we did not find between-group differences based on urgent care costs. One possible explanation is that the TSE group may experience frequent non-emergent illnesses for which they can be treated at an urgent care with low acuity-related care, resulting in treatment and costs similar to those of the non-TSE group. Urgent cares appear to be emerging as a new healthcare setting for certain pediatric subpopulations, such as those with public insurance and who underutilize primary care, who are historically considered high ED utilizers for nonurgent complaints [28, 31]. However, urgent cares may continue to be a setting for repeat visits by those with chronic conditions such as asthma [28, 32, 33]. Children presenting to the pediatric ED with TSE-related diagnoses may have higher illness severity that requires higher resource utilization (e.g., diagnostic testing) and hospital admission [34], potentially leading to higher costs.

Compared to rates of the non-TSE group, the child TSE group had higher rates of respiratory illnesses, prematurity, and TSE-related diagnoses, with the exception of conjunctivitis. These illnesses account for increased medical costs attributable to TSE in other U.S. states [35–37]. For example, previous research on mean TSE-attributable healthcare expenditures in California found the costliest diseases among children are middle ear disease ($5.6 million), asthma ($4.5 million), and respiratory infections ($754,000) or related chronic symptoms ($448,000) [35]. A prior study among the U.S. population that assessed three common acute conditions including otitis media and pharyngitis, which were prevalent among our population, found that the overall cost per episode for treatment in urgent cares was $156 compared to ED costs of $570 for the same diagnoses [29]. Another study not specific to children found mean costs per episode were $356 at an ED compared to $124 at an urgent care [30]. A study among children with Medicaid insurance found the median payments for urgent care visits ($77) were significantly lower than ED visits ($186) [23]. It is important to note that nearly three-quarters (73.2%) of children included in our study had Medicaid insurance/self-pay, which was a significant covariate across the pediatric ED and urgent care cost models. Future research should assess TSE-attributable costs distinctly in the pediatric ED and urgent care settings over time.

We found that the child TSE group had higher mean hospital admissions than the non-TSE group. After adjustment for child sociodemographics, we also report that the child TSE group had an increased risk of having hospital admissions than the non-TSE group. Other research indicated children with high biochemically validated TSE levels had an increased risk of having hospital admissions than children with no/minimal TSE levels [38]. We found that those in the child TSE group had higher rates of being admitted to the hospital during their baseline visit than the non-TSE group. However, we found no between-group differences in baseline admissions costs. There are many possible explanations for this, including the high correlation between TSE and socioeconomic status [4, 39], and more research is needed to investigate other factors. Thus, one reason may be that when compared with unexposed children, those with TSE commonly have lower socioeconomic status, as measured by having Medicaid insurance in this study, and live in households that are impoverished and experience family-related socioeconomic hardships as revealed in other studies [4, 39]. Clinicians may take these factors into consideration while making decisions about hospital admissions. For example, if a tobacco smoke-exposed child was admitted with a moderately severe illness (e.g., to monitor asthma severity), then this would result in lower resource utilization and costs compared to an unexposed child admitted with a highly severe illness (e.g., asthma patient who requires continuous nebulized Albuterol and monitoring). We report that TSE was highly related to hospital admissions, an indicator of illness severity, over the 12-month period following the baseline visit. Hospitalization may be a time to screen and identify parents who smoke and deliver smoking cessation and TSE reduction education. For example, a smoking cessation intervention for parents and patients, in which 55% of children were critically ill and in the intensive care unit, found that offering programming during this time was a good starting point to receive cessation resources and begin quitting smoking [40].

This study was limited by the use of EMRs from one Midwestern hospital’s database. However, the vast majority of pediatric ED and urgent care patients regularly seek emergency care at this hospital instead of other settings (e.g., adult hospitals). We speculate that we captured most of their acute healthcare utilization data over the 12-months. We limited our study to pediatric ED visits, urgent care visits, and hospitalizations, and did not assess doctor’s office visits. This may have impacted our results since patients may have had non-acute office visits for preventive care 12-months following their baseline pediatric ED and urgent care visits that were not accounted for in this study. Pediatric patients with more health concerns may use more outpatient services, and future TSE research should include these visits. Further, we had varying inclusion criteria where the child TSE group lived with a smoker and presented with a TSE-related chief complaint whereas the non-TSE group did not live with a smoker and presented with a wide range of chief complaints. We included children from the TSE group in a consecutive manner based on their RCT enrollment, and then matched the children in the TSE group with the non-TSE group using propensity scores. We did not biochemically verify group membership, but were able to find differences using the ratio of one TSE group child to three non-TSE group children. This decreases the possibility that many children in the non-TSE group had underreported exposure. We were unable to assess exclusive e-cigarette aerosol exposure since none of the parents of children in the TSE group exclusively used these non-combustible tobacco products due to study inclusion/exclusion criteria. Only about 5% of parents concurrently used e-cigarettes with combustible tobacco products in the present study, which aligns with our RCT population’s current use rates [41]. Research is needed to assess the relationship between exclusive e-cigarette aerosol exposure and healthcare utilization patterns among children. Additionally, data on whether children were exposed to tobacco products while in utero and the amount of exposure were not available. Thus, we were unable to assess the potential confounding of prenatal TSE. Cost data were cross-sectional in nature and only assessed from billed charges incurred during baseline visits and hospitalizations. Additional research is needed to assess cost data over time.

## Conclusions

This study found that tobacco smoke-exposed children had higher urgent care utilization and hospital admissions over 12-months and pediatric ED costs at baseline relative to unexposed children. Given higher urgent care visits and higher pediatric ED costs for children with TSE coupled with the fast-paced nature of these visits, acute care settings may best serve as locations in which brief parental smoking cessation and child TSE reduction counseling is initiated using integrated EMR prompts, followed by the delivery of resources and referrals to free cessation services such as the tobacco Quitline [41–43]. If the patient is hospitalized or referred to their primary care physician, additional counseling could be provided. Tobacco control interventions initially offered in the pediatric ED and urgent care settings may result in the reduction of child TSE, related illnesses, and related healthcare costs.
